# Classification models for assessing coronary artery disease instances using clinical and biometric data: an explainable man-in-the-loop approach

**DOI:** 10.1038/s41598-023-33500-9

**Published:** 2023-04-24

**Authors:** Agorastos-Dimitrios Samaras, Serafeim Moustakidis, Ioannis D. Apostolopoulos, Nikolaos Papandrianos, Elpiniki Papageorgiou

**Affiliations:** 1 Department of Energy Systems, University of Thessaly, Larisa, Greece; 2 Department of Medical Physics, School of Medicine, University of Patras, Patras, Greece; 3 AIDEAS OÜ, Tallinn, Estonia

**Keywords:** Cardiology, Risk factors, Signs and symptoms, Biomedical engineering

## Abstract

The main goal driving this work is to develop computer-aided classification models relying on clinical data to identify coronary artery disease (CAD) instances with high accuracy while incorporating the expert’s opinion as input, making it a "man-in-the-loop" approach. CAD is traditionally diagnosed in a definite manner by Invasive Coronary Angiography (ICA). A dataset was created using biometric and clinical data from 571 patients (21 total features, 43% ICA-confirmed CAD instances) along with the expert’s diagnostic yield. Five machine learning classification algorithms were applied to the dataset. For the selection of the best feature set for each algorithm, three different parameter selection algorithms were used. Each ML model’s performance was evaluated using common metrics, and the best resulting feature set for each is presented. A stratified ten-fold validation was used for the performance evaluation. This procedure was run both using the assessments of experts/doctors as input and without them. The significance of this paper lies in its innovative approach of incorporating the expert's opinion as input in the classification process, making it a "man-in-the-loop" approach. This approach not only increases the accuracy of the models but also provides an added layer of explainability and transparency, allowing for greater trust and confidence in the results. Maximum achievable accuracy, sensitivity, and specificity are 83.02%, 90.32%, and 85.49% when using the expert's diagnosis as input, compared to 78.29%, 76.61%, and 86.07% without the expert's diagnosis. The results of this study demonstrate the potential for this approach to improve the diagnosis of CAD and highlight the importance of considering the role of human expertise in the development of computer-aided classification models.

## Introduction

In modern society, cardiovascular diseases (CVD) are one of the most common health problems and the leading cause of death^1^. According to the World Health Organization (WHO), an estimated 17.9 million people died from CVDs in 2019, representing 32% of all global deaths^2^. Coronary Artery Disease (CAD) is the most common type of cardiovascular disease. It is thus safe to assume that timely diagnosis and treatment of CAD can lead to a significant decrease in global mortality. This work aims to introduce a new tool based on Machine Learning (ML) classification models meant to be used in a consultative manner by experts in the process of diagnosis.

Single Photon Emission Computed Tomography (SPECT) and Positron Emission Tomography (PET) are the main tools used for the visual assessment of CAD. Experts visually interpret these images and, by combining clinical, historical, and biometric data, conclude on a verdict and its consequent treatment. On the other hand, recently, Artificial Intelligence (AI) has significantly supported healthcare intervention decisions via customized Medical Decision Support Systems (MDSS). A repertoire of predictive models based on boosted trees, random forests, and straightforward Deep Learning (DL) have provided highly accurate predictions for multi-parameter and complex designs in medicine^3^. Imaging technologies and clinical data do not ensure a definite diagnosis, whilst there are many situations that pose diagnostic dilemmas. Therefore, it is very often that a patient is subjected to ICA, invasive diagnosis, and treatment of CAD. Ideally, for healthy subjects, this procedure should be avoided.

There is a plethora of related work^4–10^ where common ML algorithms are used to predict CAD instances with varying results, ranging accuracy-wise from 71.1% to over 98% when also employing image data. Commonly, studies with datasets exceeding 200 patients tend to achieve accuracy lower than 84%^4^. These studies use both clinical data and image data (mainly from SPECT and PET) as inputs to predict CAD. In Ref.^5^, GA is used to select a feature set, which is then passed to Support Vector Machine (SVM) algorithms. In Ref.^6^, SPECT image data from a dataset of 192 patients is used along with two different classification models. Researchers in ^9^ also did a 5-year follow-up of 10,030 patients with suspected coronary artery disease and then compared the prediction results of ML combining clinical and CCTA data against existing clinical or CCTA metrics alone; ML was found to significantly better predict 5-year ACM. Table 1 depicts a summary of recent work specifically on CAD classification. Further discussion and comparison with the findings of the current work is provided in the “Discussion” section.

**Table 1:** Related Work on CAD classification using clinical data.

References	Dataset Size	Data Types	Results	Remarks
Apostolopoulos et al.^11^	Train Data: 303 Test Data: 303	demographic, medical tests, symptoms, patient history	Accuracy: 85.47% Sensitivity: 89.3% Specificity: 79.31% AUC: 82.45%	Doctor not included
Apostolopoulos et al.^12^	Train Data: 566 Test Data: 566	demographic, medical tests, symptoms, patient history	Accuracy: 75.79% Sensitivity: 74.07% Specificity: 76.16% AUC: 71.09%	Doctor not included
Apostolopoulos et al.^13^	Train Data: 303 Test Data: 303	demographic, medical tests, symptoms, patient history	Accuracy: 78.21% Sensitivity: 83.95% Specificity: 68.96%	Doctor not included
Alizadehsani et al.^14^	Train Data: 500 Test Data: 500	demographic, medical tests, symptoms, lab results	Best of all: SVM: Accuracy: 96.4% Sensitivity: 84.6% Specificity: 48.4%	Doctor not included
Muhammad et al.^15^	Train Data: 405 Test Data: 101	demographic, medical tests, symptoms,	Best of all: Random forest Accuracy: 92.04% Sensitivity: 86.5% Specificity: 83.34%	Doctor is the reference
Sayadi et al.^16^	Train Data: 303 Test Data: 303	demographic, medical tests, symptoms, lab results	Best of all: SVM: Accuracy: 95.45% Sensitivity: 95.91% Specificity: 91.66%	Doctor not included
Liu et al.^17^	Train Data: 185 Test Data: 185	demographic, medical tests,	CAD Sensitivity: 80.97% ± 7.75% Specificity: 61.37% ± 14.4% Non-CAD Sensitivity: 61.37% Specificity:80.97%	Doctor not included
Xiao et al.^18^	Train Data: 209 Test Data: N/A	demographic, medical tests, symptoms	Accuracy: 89.5% Sensitivity: 89.8% Specificity: 88.9%	Doctor not included
Johri et al.^19^	Train Data: 500 Test Data: 500	demographic, medical tests, patient history	Best of all: LSTM Accuracy: 95.34% AUC: 0.99"	Doctor not included
Benjamins et al.^20^	Train Data: 830 Test Data: 830	demographic, medical tests, expert’s yield, symptoms	Expert AUC: 0.87 LogitBoost AUC: 0.91	Doctor-in-the-loop

In the scientific pool of relevant work, despite the diversity of input data and classification mechanisms, one essential input factor was almost never utilized: the expert's judgment. Typically, while treating patients with suspected CAD, well-trained clinicians determine if the patient is at risk for CAD or is healthy. The majority of these specialists are medical school graduates with years of practical experience in cardiac disease. Consequently, their conclusion reflects their academic background and years of practical expertise. Additionally, research suggests that having human/doctor-in-the-loop can be beneficial to the overall performance of ML or automated systems^21,22^. Therefore, this work uses the expert’s verdict as an additional input datum and tries to build upon it and enhance it. Another differentiating point is that different feature selection algorithms were used to select the most accurate feature set, and then the results were evaluated using stratified tenfold cross-validation^23^. All in all, the four major contributions of our work can be summed up as follows: (1) the use of expert’s prediction in the feature pool and highlighting its importance by comparing results without it; (2) assessing the efficiency of feature selection based on GA and forwards/backwards SFS, (3) studying the performance of five highly used ML algorithms on the subject with the help of five different metrics and tenfold cross validation; and finally (4) proposing the optimal feature sub-set and the accompanying best-performing ML algorithm to be used in a computer-aided decision-making system for CAD diagnosis.

The organization of the rest of this paper is as follows. “Methods” section outlines the details of the patient dataset, including the characteristics and demographics of the participants, as well as the ML algorithms employed for classification. The evaluation process used to measure the performance of the models is also outlined in this section, along with the proposed explainability analysis. In “Results” section, the experimental results are presented, including the performance metrics and any notable findings. “Discussion” section delves deeper into the strengths and weaknesses of the proposed models, comparing them to previous works in the field. Finally, “Conclusion” section concludes the study by summarizing the key findings and suggesting potential avenues for future research.

## Methods

### Patient population

This study involves 571 participants. Of this pool, 248 patients were ICA-confirmed CAD positive (43.43%) and the rest were healthy. There was variation in both the biometric and clinical information of the subjects. More specifically on the demographic side, 79.68% were male, the ages ranged from 32 to 90, and the Body Mass Index (BMI) from 16.53 (underweight) to 87.2 (extremely obese). On the other hand, a variation of clinical data was used (e.g. Dyslipidemia, Diabetes etc.) in combination with some historical data (e.g. smoker/non-smoker, family history of CAD etc.). The features are presented in Table 2.

**Table 2:** Features used as input by prediction models, after binary normalization.

A/A	Feature Name	Description	Feature Class/Type
1	known Cad	Coronary Artery Disease	Predisposing Factor
2	previous AMI	Acute Myocardial infarction	Predisposing Factor
3	previous PCI	Percutaneous Coronary Intervention	Predisposing Factor
4	previous CABG	Coronary artery bypass graft surgery	Predisposing Factor
5	previous Stroke	Stroke	Predisposing Factor
6	Diabetes	Diabetes positive patient	Predisposing Factor
7	Smoking	Smoker/Non-smoker	Predisposing Factor
8	Arterial Hypertension	Known Arterial Hypertension instance	Recurrent Diseases
9	Dyslipidemia	Known Dyslipidemia instance	Recurrent Diseases
10	Angiopathy	Known Angiopathy instance	Recurrent Diseases
11	Chronic Kidney Disease	Known Chronic Kidney Disease instance	Recurrent Diseases
12	Family History of CAD	CAD occurrence in family	Recurrent Diseases
13	Asymptomatic	No symptoms	Symptoms
14	Atypical	Atypical symptoms	Symptoms
15	Angina-like	Angina-like symptoms	Symptoms
16	Dyspnea	Dyspnea on exertion	Symptoms
17	Precordial Pain	Precordial pain occurrence	Symptoms
18	Sex	Male/female	Demographics
19	Normal Weight	BMI lower than 24.9	Demographics
20	Overweight	BMI between 25 to 29.9	Demographics
21	Obese	BMI over 30	Demographics
22	< 40	Aged under 40	Demographics
23	40–50	Aged between 40 to 50	Demographics
24	50–60	Aged between 50 to 60	Demographics
25	> 60	Aged over 60	Demographics
26	RST ECG	Diagnostic Resting Electrocardiogram	Diagnostic Test (ECG)
27	Doctor	Expert’s prediction	Expert’s Prediction
28	CAD	ICA—ground truth	Reference Variable

The patients comprising this work’s dataset underwent gated-SPECT Myocardial Perfusion Imaging (MPI) and were subsequently subjected to ICA within 60 days from MPI for further investigation. This is the state-of-the-art procedure to determine whether a patient is actually CAD-affected and this was used as the ground truth for this work.

The patient data have been recorded at the Clinical Sector of the Department of Nuclear Medicine of the University Hospital of Patras from 16/2/2018 to 28/02/2022. Data collection has been approved by the ethical committee of the University General Hospital of Patras (Ethical and Research Committee of the University Hospital of Patras, protocol number 108/10-3-2022) and the requirement to obtain informed consent was waived by the Director of the Diagnostic Center of the University due to its retrospective nature. The retrospective nature of the study waives the requirement to obtain informed consent from the participants. All data-related processes were performed anonymously. All procedures in this study were in accordance with the Declaration of Helsinki.

### Dataset

Binary encoding was the most suitable method to represent the majority of features, which were binary in nature. For instance, patients were classified as having diabetes or not, or as male or female. Nevertheless, there were two continuous data fields: age and BMI. Hence, some data transformation was needed to be applied to these two categories for their data to fit the binary problem. For the normalization of age, we split up the information into 4 different fields. As is widely known in the medical field, the most common years for CAD to occur are between 40 and 60 years of age^24^. Even though inside this range there is great variation in symptoms, for humans younger than 40 or older than 60, age is not a deciding or indicating factor as far as CAD is concerned^25^. So, the 4 different fields used for age are < 40, 40–50, 50–60 and > 60. On the matter of BMI, our approach was the one adopted by WHO^26^. Thus, the BMI information was divided into 3 mutually exclusive fields; underweight, normal weight, and obese. Further categorization of obesity (i.e. moderately obese, severely obese and very severely obese) was not used, as it does not offer much differentiation for CAD instance purposes^27^.

Overall, after the data preprocessing was done, the information fueling this work is contained in 28 features, as shown in Table 2. These can be categorized in seven groups:

(i) Predisposing Factors (feature No 1–7)
(ii) Recurrent Diseases (feature No 8–12)
(iii) Symptoms (feature No 13–17)
(iv) Demographics (feature No 18–25)
(v) Diagnostic Test (ECG) (feature No 26)
(vi) Expert’s Prediction
(vii) Invasive Coronary Angiography (the actual reference)

The predictions of the ML models were tested against the ICA results. Invasive coronary angiography uses X-rays to examine blood flow to the heart, most commonly for patients presenting with acute coronary syndromes, or heart attacks.

### Feature selection

The initial phase consisted of filtering the original sum of 26 features (excluding the ground truth field—‘CAD’) to highlight the most significant ones, hence optimizing the accuracy of the prediction algorithms. In order to achieve that, we used 3 very common feature selection algorithms, forwards SFS, backwards SFS and Genetic Algorithm^28–31^. In a loop of 5 iterations, they were applied to each of the ML algorithms studied in this paper. Each iteration loop resulted in a feature subset and a corresponding accuracy metric score. This function produced a feature subset and an accompanying accuracy score for each feature selection algorithm and each ML algorithm. So for every ML algorithm, we had 3 selected feature subsets with their relative scores. Eventually, the best one was selected to be used in the training, prediction, and evaluation procedures. The aforementioned steps are shown in Algorithm 1.

Particularly for the genetic algorithm, which allows for more complex parameter customization than the SFS algorithms, numerous configurations were evaluated. Each configuration had different parameters (e.g., crossover probability, number of generations, etc.) and produced a different subset for every ML algorithm. Each ML algorithm’s particular subsets were evaluated separately. Afterwards, the best of them (accuracy-wise) was compared with the most accurate subsets produced by the SFS feature selection algorithms in order to determine the best feature subset for the particular ML algorithm, which was to be used in the next step of the process.
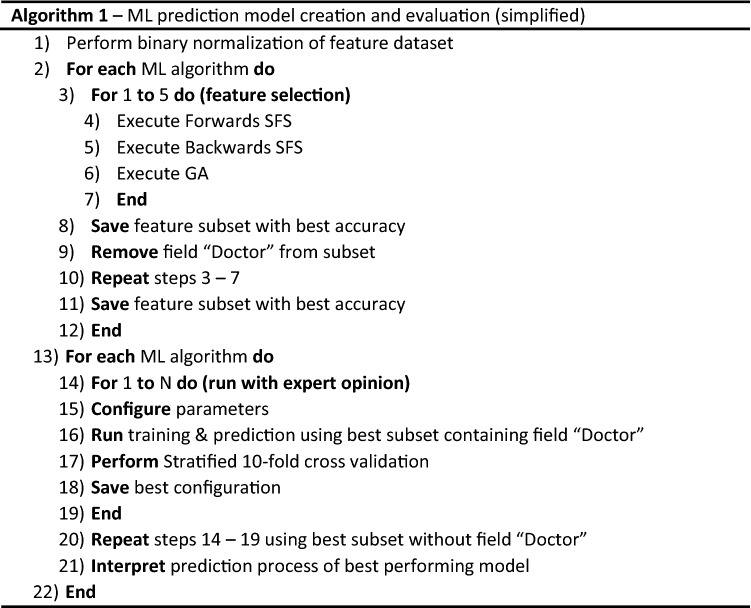


### ML algorithms

Support Vector Machine, Decision Trees, K Nearest Neighbors, Adaptive Boosting and Random Forest are the five well-documented ML classification algorithms used in this study. This range of algorithms has been widely used by the research community^4,15,32–37^ with proven effectiveness.

The SVM classifier^38^ is a supervised machine learning algorithm developed by Vladimir Vapnik and his colleagues at AT&T Bell Laboratories. It projects training data into a higher dimensional space via mappings known as kernels. Then a boundary (also known as hyperplane) is devised with the criterion of maximum separation between classes. New examples are then projected to the hyperplane, and the boundary is used to determine classification labels.

As far as tree-based ML algorithms go, Decision Tree^39^ is the simplest form. It functions by constructing a tree structure in a recursive fashion (hence the name). The classification is performed by assigning every sample to leaf nodes (decision splits) accordingly. It is also the basis for more complex tree-based ML algorithms, such as Random Forests or Boosted Forests.

The working theory behind K-nearest neighbor^40^ revolves around the k parameter. This parameter is used to assign the number of nearest neighbors that are tested for similarity to the new input with the use of a distance function. For instance, for k = 2 the algorithm assigns to a new instance the label that minimizes its distance from two of its neighbors.

Adaptive Boosting^41^, or as it is commonly known, AdaBoost, is a classification ML algorithm designed by Yoav Freund and Robert Schapire in 1995. It is most often applied in cases of binary classification. AdaBoost uses a form of “adaptive learning” where the output of learning algorithms (“weak learners”) is combined into a weighted sum that represents the final output of the boosted classifier. Subsequent weak learners are tweaked in favor of those instances misclassified by previous classifiers. The most common use configuration of AdaBoost employs Decision Trees as weak learners. This algorithm is adaptive in the sense that subsequent weak learners are tweaked in favor of those instances misclassified by previous classifiers. In some problems, it can be less susceptible to the overfitting problem than other learning algorithms. Individual learners can be weak, but as long as the performance of each one is slightly better than random guessing, the final model can be proven to converge to a strong learner.

Random forest^42^ is another tree-based supervised ML algorithm. At its core, it consists of multiple decision trees, and it is used mainly for regression and classification problems. The individual decision trees function as a group, where each tree produces a label/class of prediction. The label chosen by the majority of trees is then selected as the model for prediction.

### Results evaluation

The final step of the model in this work is to evaluate the best prediction models proposed by each of the ML classifiers and eventually choose the best one. To achieve this, we used 6 widely implemented metrics, accuracy, sensitivity, specificity, Jaccard score, F1-score and confusion matrix. All of the above metrics determine the performance of a model by using a combination of correlation between True Positive (TP), True Negative (TN), False Positive (FP) and False Negative (FN) instances.

Accuracy may be the most frequently employed parameter for evaluating the prediction performance of an ML model. It determines the percentage of correctly predicted instances over the total number of cases. It is expressed as follows:

1 $$Accuracy=\frac{TP+TN}{\mathrm{TP}+\mathrm{TN}+\mathrm{FP}+\mathrm{FN}}$$

Sensitivity focuses on the percentage of CAD instances correctly predicted by the ML algorithms. This metric expresses which of all the CAD positive instances were actually correctly predicted by the model, and it is expressed using the following equation:

2 $$\mathrm{Sensitivity}=\frac{TP}{\mathrm{TP}+\mathrm{FN}}$$

Specificity, on the other hand, expresses the percentage of healthy instances correctly predicted by the ML algorithms. This metric is used to indicate which of all the CAD negative instances were actually correctly predicted by the model, and it is implemented as follows:

3 $$\mathrm{Specificity }=\frac{TN}{\mathrm{TN}+\mathrm{FP}}$$

The Jaccard Index, or Jaccard similarity coefficient, is a statistic used to showcase the similarity degree between sample sets. The measurement emphasizes similarity between finite sample sets, and is formally defined as the size of the intersection divided by the size of the union of the sample sets. In this particular case, it is expressed mathematically as:

4 $$\text{Jaccard index }=\frac{TP}{\mathrm{TP}+\mathrm{FP}+\mathrm{FN}}$$

F1-score, also called F-score, is employed mainly in binary classification cases and is a way of combining the precision and recall of a model. It is defined as the harmonic mean of the model’s precision (number of true positive results divided by the number of all positive results, including FP) and recall (number of true positive results divided by the number of all samples that should have been identified as positive). Its mathematic formula is:

5 $${\mathrm{F}}_{1} =\frac{2\times TP}{2\times \mathrm{TP}+\mathrm{FP}+\mathrm{FN}}$$

Lastly, a Confusion Matrix is a summary of prediction results on a classification problem. In binary classification problems, it is a 2 by 2 matrix, which is formed by the TP, TN, FP and FN fields. Such a confusion matrix has the following form:

**Table:** 

		Predicted condition	
		Positive (PP)	Negative (PN)
Actual condition	Positive (P)	True positive (TP)	False negative (FN)
	Negative (N)	False positive (FP)	True negative (TN)

The prediction results of each of the ML algorithms were passed through a tenfold stratified cross validation. Then the cross validated results were used to calculate the evaluation metrics for each ML model. These are presented in Tables 3 and 4 of the “Results” section.

**Table 3:** Best accuracy for each ML model—with expert’s opinion as input. Standard deviation (STD) is included in parentheses.

	SVM	DT	KNN	ADA	RF
GA	**82.66%** (3.80)	**81.96%** (4.95)	**83.01%** (6.34)	**81.26%** (4.13)	**83.02%** (4.27)
Bwd SFS	82.13% (4.15)	78.63% (6.34)	82.66% (6.87)	79.69% (4.74)	75.48% (5.13)
Fwd SFS	81.78% (5.28)	78.81% (5.80)	82.14% (5.65)	80.74% (5.15)	77.23% (3.96)
All features	**80.73%** (5.09)	71.10% (5.30)	79.34% (4.01)	77.60% (5.65)	77.23% (5.96)

Significant values are in bold.

**Table 4:** Best accuracy for each ML model—without expert’s opinion as input. STD is included in parentheses.

	SVM	DT	KNN	ADA	RF
GA	78.12% (4.24)	73.04% (2.49)	76.37% (3.10)	**76.54%** (3.74)	**77.59%** (3.13)
Bwd SFS	77.77% (3.61)	76.01% (4.32)	**77.95%** (3.38)	75.49% (4.12)	74.09% (3.72)
Fwd SFS	**78.29%** (3.86)	**78.29%** (4.32)	75.84% (4.44)	75.49% (3.82)	76.54% (2.62)
All features	**74.79%** (2.19)	66.37% (5.73)	70.76% (3.45)	74.09% (4.02)	72.33% (6.15)

Significant values are in bold.

The same procedure was run in 3 test scenarios:

(i) With feature selection and using the expert’s verdict
(ii) Without feature selection and using the expert’s verdict
(iii) With feature selection and without using the expert’s verdict

The expert’s verdict has an accuracy of 78.81%. This prediction is based on all the clinical data, image data from SPECT/PET and years of theoretical knowledge and practical expertise. This accuracy is what this work aims to enhance and will thus be used as a soft threshold to determine the performance of each AI model. Needless to say, any model that uses the expert’s verdict as input and does not achieve an accuracy metric score higher than 78.81% will not be considered.

### Results interpretation

In conclusion, the best-performing prediction model will be examined to provide an explanation for its results. This will provide an added layer of transparency and understanding of the model's decision-making process, making it more trustworthy and reliable for users.

The majority of AI systems typically operate as black boxes. This obviously decreases the level of confidence, especially when unexpected outputs are predicted. Therefore, by making the ML prediction tool of this study more comprehensible and shedding light on its prediction making process, we would be able to make it more appealing and trustable. In order to achieve that, we employ mainly two interpretability mechanisms: Cohen effect size and SHAP values.

The Cohen effect size is a statistical measure used to determine the magnitude of the difference between two groups. It is calculated by dividing the difference between the means of the groups by the pooled standard deviation. Cohen^43^ proposed that an effect size of 0.2 should be considered small, 0.5 as medium, and 0.8 as large. This measure is commonly used in various fields (e.g., psychology, medicine, etc.) to interpret the results of experiments and studies. It is a useful tool for determining the practical significance of the results and comparing them to previous research.

On the other hand, SHAP analysis^44^ is one of the most common techniques used to increase the transparency of an ML prediction model. Using cooperative game theory concepts, this type of analysis tackles the explainability problem, by treating each feature as a “player” in a game where the prediction is the goal. The method tries to split the goal among all the features in a fair manner or, alternatively, assign each feature an importance value for a particular prediction, depending on how much each feature contributed to the final score/goal.

## Results

The steps outlined in Algorithm 1, as presented in the “Methods” section, were executed in a Linux environment consisting of an 8-core i7 CPU, 16 GB DDR3 RAM, and Ubuntu 20.04LTS. The core coding for this project was developed using the Python programming language. Several machine learning-specific Python libraries, such as sklearn, shap, and genetic_selection, were particularly useful in creating an automated environment capable of running and evaluating the performance of multiple machine learning prediction models.

SVM, decision trees (DT), k nearest neighbors (KNN), adaptive boosting (ADA), and random forest (RF) are five well-documented ML classification algorithms that were used in this work. To minimize the dimensionality of the initial feature space for each ML model, evolutionary algorithms (genetic algorithm—GA), forward feature selection (SFS), and backward feature selection (SFS) techniques were utilized. The training and evaluation procedures were repeated for two scenarios: with and without the doctor's diagnosis as input.

### Feature selection impact

Table 3 showcases the best accuracy achieved by each ML model and which feature selection algorithm led to the relative data subset when including the field “Doctor” (the expert’s diagnosis). The best testing accuracy was achieved by the RF-based ML model with a feature subset selected using GA. For comparison, the last row showcases how each model performed on the dataset without feature selection. The SVM model had the highest testing accuracy at 80.73% (which is 1.92% higher than the average expert's opinion).

On the other hand, Table 4 depicts the best accuracy achieved by each ML model and which feature selection algorithm led to the relative data subset without considering as input the field “Doctor” (the expert’s diagnosis). In this testing scenario, SVM and DT both achieved the best accuracy. Additionally, Forwards SFS gave the optimal feature subsets for both models. For comparison, the last row showcases how each model performed on the dataset without feature selection. The SVM model once again achieved the best predicting accuracy at 74.79% (which is, though, 4.02% lower than the average expert's opinion).

### Results using subsets from feature selection, including expert’s verdict

After running feature selection to improve the dataset beforehand, the performance of all algorithms improved dramatically. For this type of tests, the results are shown in Table 5. All ML models attained an accuracy greater than 78.81% (the expert’s accuracy). Specifically, the Random Forest model had the highest accuracy (83.02%) and specificity (85.49%), whereas the SVM model had the highest results for all other metric scores. In addition, Table 6 demonstrates the highest performing feature subsets selected for each ML method.

**Table 5:** Metrics scores achieved on selected feature subsets including expert’s verdict. STD is included in parentheses.

*w Doctor*	SVM	DT	KNN	ADA	RF
sensitivity	**88.71%** (4.01)	86.29% (5.74)	83.47% (7.92)	81.85% (7.41)	83.06% (6.42)
specificity	84.52% (6.03)	82.04% (6.74)	84.21% (7.45)	82.04% (7.22)	**85.49%** (5.27)
accuracy	82.66% (3.72)	81.96% (4.95)	83.01% (6.34)	81.62% (4.74)	**83.02%** (4.27)
Jaccard	**73.83%** (4.86)	69.93% (7.20)	69.23% (9.66)	63.34% (7.21)	69.83% (6.87)
f1	**86.39%** (3.49)	83.95% (5.67)	83.92% (6.76)	82.00% (5.15)	84.43% (4.84)

Significant values are in bold.

**Table 6:** Best performing feature subsets for each ML model and their selection algorithm (with expert verdict).

ML	Subset	Algorithm
SVM	['known CAD', 'previous PCI', 'previous CABG', 'Diabetes', 'Smoking', 'Dyslipidemia', 'Angiopathy', 'Chronic Kidney Disease', 'ASYMPTOMATIC', 'ATYPICAL SYMPTOMS', 'ANGINA LIKE', 'RST ECG', 'male', '40–50', 'Doctor: Healthy']	GA
DT	['previous AMI', 'previous CABG', 'Arterial Hypertension', 'Angiopathy', 'Chronic Kidney Disease', 'Family History of CAD', 'ANGINA LIKE', 'male', ' < 40', 'Doctor']	GA
KNN	['known CAD', 'previous AMI', 'previous CABG', 'Diabetes', 'Smoking', 'Arterial Hypertension', 'Dyslipidemia', 'Angiopathy', 'Chronic Kidney Disease', 'ASYMPTOMATIC', 'ATYPICAL SYMPTOMS', 'ANGINA LIKE', 'INCIDENT OF PRECORDIAL PAIN', 'male', 'Overweight', ' < 40', '50–60', 'Doctor']	GA
ADA	['known CAD', 'previous AMI', 'Diabetes', 'Family History of CAD', 'ATYPICAL SYMPTOMS', 'ANGINA LIKE', 'INCIDENT OF PRECORDIAL PAIN', 'RST ECG', 'male', 'Overweight', 'Obese', ' < 40', 'Doctor: Healthy']	GA
RF	['known CAD', 'previous PCI', 'Diabetes', 'Chronic Kidney Disease', 'ANGINA LIKE', 'RST ECG', 'male', ' < 40', 'Doctor']	GA

### Results using subsets from feature selection, without expert’s verdict

The final batch of results is displayed in Tables 7 and 8. With expert verdict no longer available as data input, the performance of every machine learning system decreased significantly. None of the models attained a better degree of accuracy than 78.81% which is the expert’s accuracy (soft threshold). The model with the best metric performance was the one that utilized the DT algorithm. It performed the best across all metrics. Table 8 displays the selected subset of features for each model without expert’s verdict.

**Table 7:** Metrics scores achieved on selected feature subsets without expert’s verdict. STD is included in parentheses.

*w/o Doctor*	SVM	DT	KNN	ADA	RF
Sensitivity	76.21% (9.30)	**76.61%** (5.74)	65.73% (9.66)	70.56% (7.85)	75.81% (8.49)
Specificity	80.80% (4.93)	**86.07%** (7.14)	84.83% (4.94)	82.04% (4.17)	82.04% (5.88)
Accuracy	**78.29%** (3.86)	**78.29%** (4.17)	76.89% (4.42)	76.54% (3.74)	77.59% (3.13)
Jaccard	60.97% (6.52)	**64.85%** (7.55)	54.88% (8.04)	57.19% (6.34)	61.44% (5.23)
f1	78.82% (4.98)	**81.90%** (7.14)	76.23% (6.67)	76.96% (4.90)	79.32% (4.09)

Significant values are in bold.

**Table 8:** Best Performing Feature Subsets for each ML model and their Selection algorithm (without expert’s verdict).

ML	Subset	Algorithm
SVM	['known CAD', 'previous PCI', 'Diabetes', 'INCIDENT OF PRECORDIAL PAIN', 'RST ECG', 'male', '40–50']	Fwd SFS
DT	['known CAD', 'previous PCI', 'previous STROKE', 'Diabetes', 'Family History of CAD', 'DYSPNOEA ON EXERTION', 'INCIDENT OF PRECORDIAL PAIN', 'RST ECG', 'male', ' < 40', '40–50']	Fwd SFS
KNN	['known CAD', 'previous CABG', 'Diabetes', 'Angiopathy', 'Chronic Kidney Disease', 'ATYPICAL SYMPTOMS', 'INCIDENT OF PRECORDIAL PAIN', 'male', '40–50', '50–60', ' > 60']	Fwd SFS
ADA	['known CAD', 'Diabetes', 'Angiopathy', 'Family History of CAD', 'ATYPICAL SYMPTOMS', 'male', '40–50']	GA
RF	['known CAD', 'previous PCI', 'Diabetes', 'INCIDENT OF PRECORDIAL PAIN', 'RST ECG', 'male', '40–50']	GA

### Interpretation of best performing model

**Figure 1:**
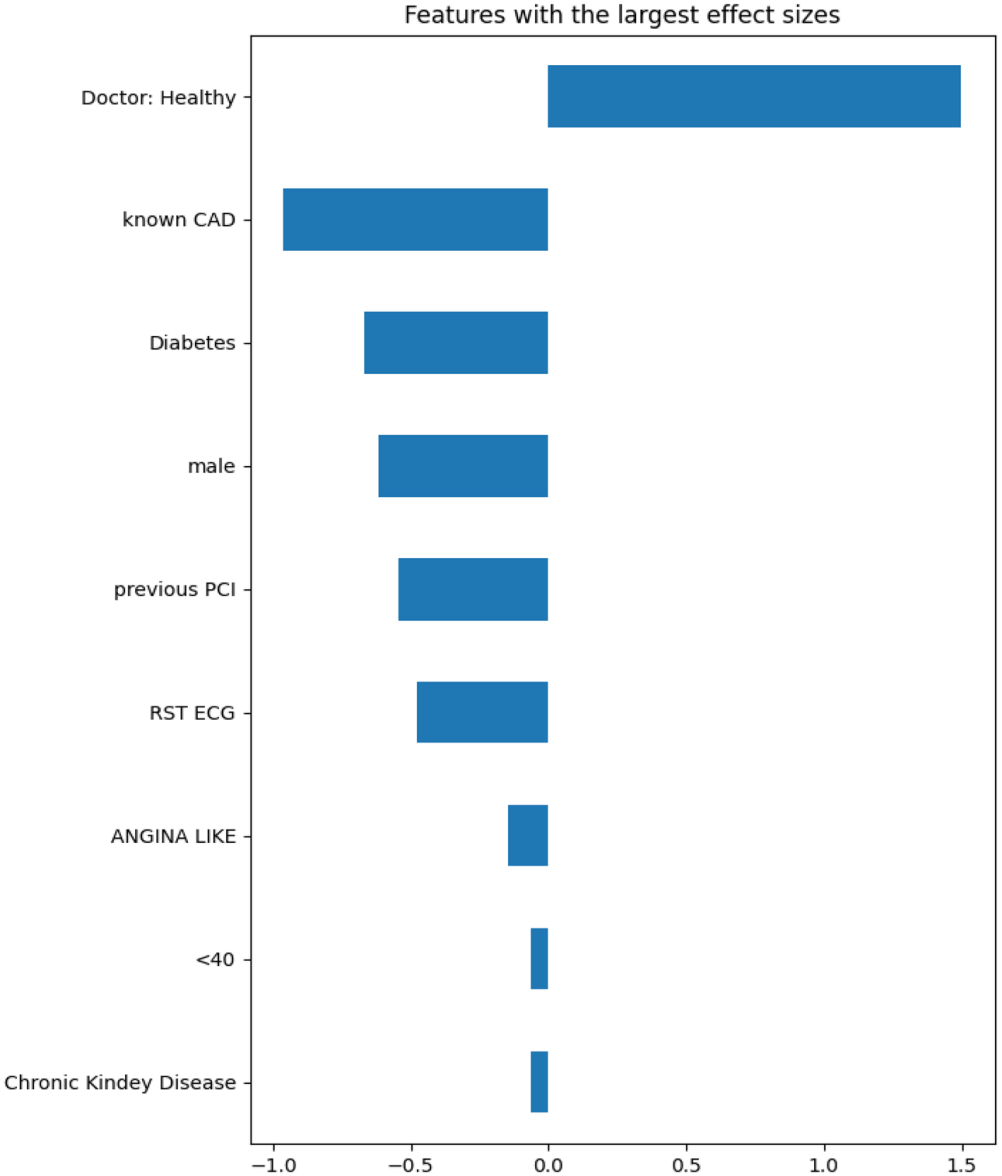
Cohen effect size for each input feature sorted by magnitude of effect.

**Figure 2:**
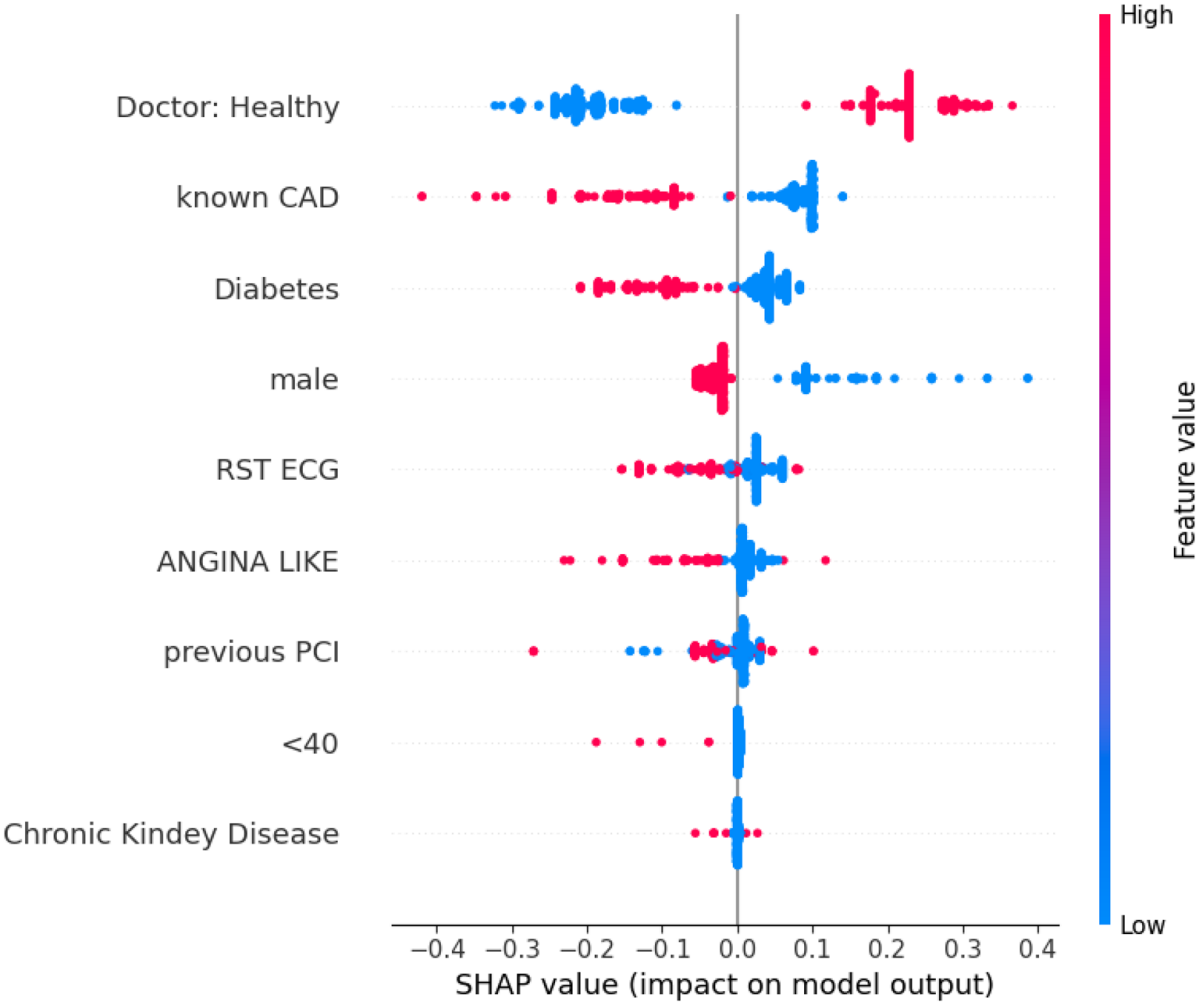
Summary plot illustrating the relative importance of each input feature in the prediction model, as represented by the SHAP values. Being a binary classification problem, feature values can either be ‘0’ or ‘1’, with value ‘1’ indicating the positive (e.g. male = 1, means the patient is male).

**Figure 3:**
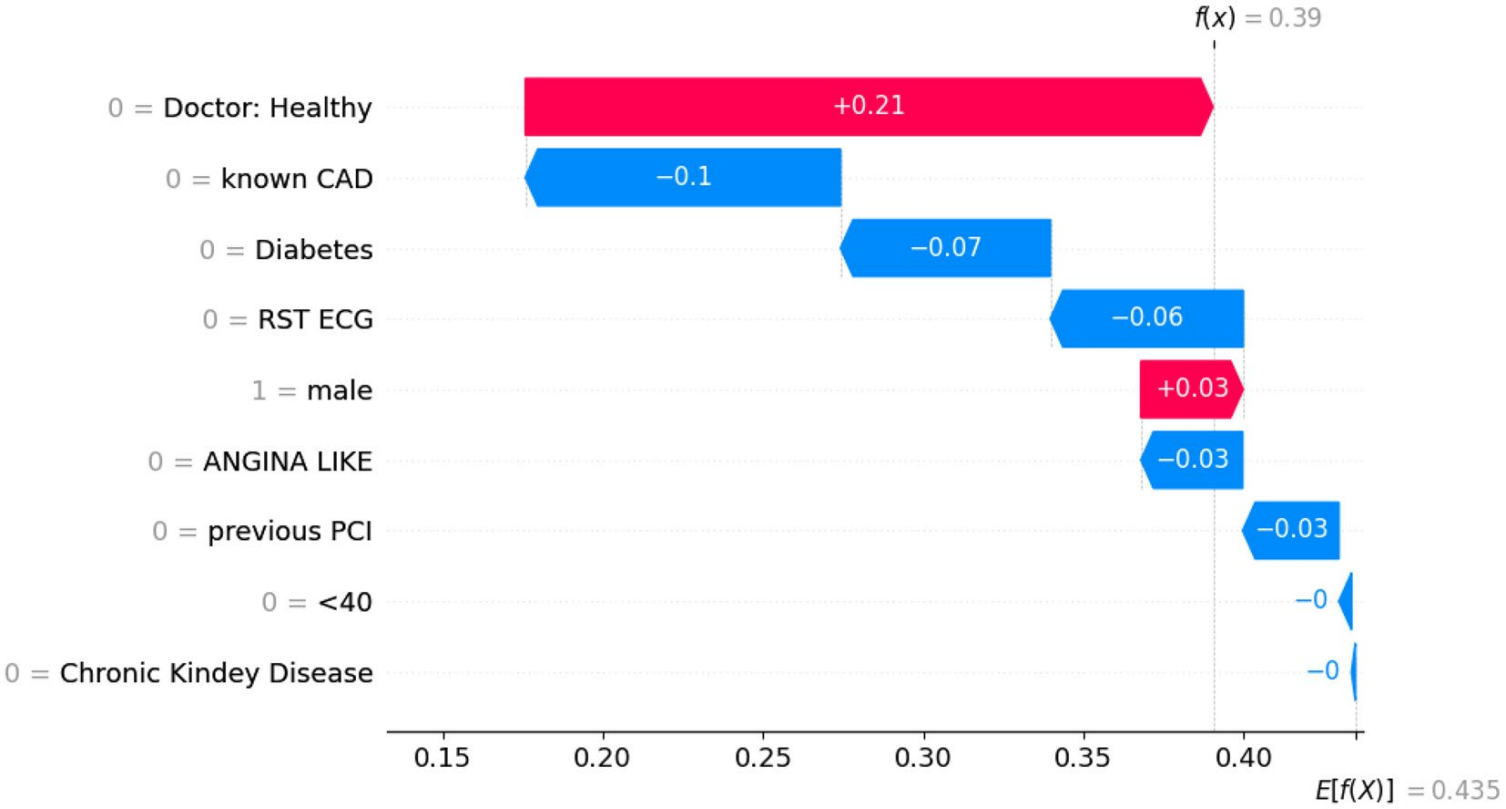
Waterfall diagram for a prediction of a healthy patient. This diagram indicates which features drove the algorithm to predict the entry as healthy. It is noteworthy, that the expert had initially wrongly identified this as a CAD instance, but ICA proved the patient was healthy.

**Figure 4:**
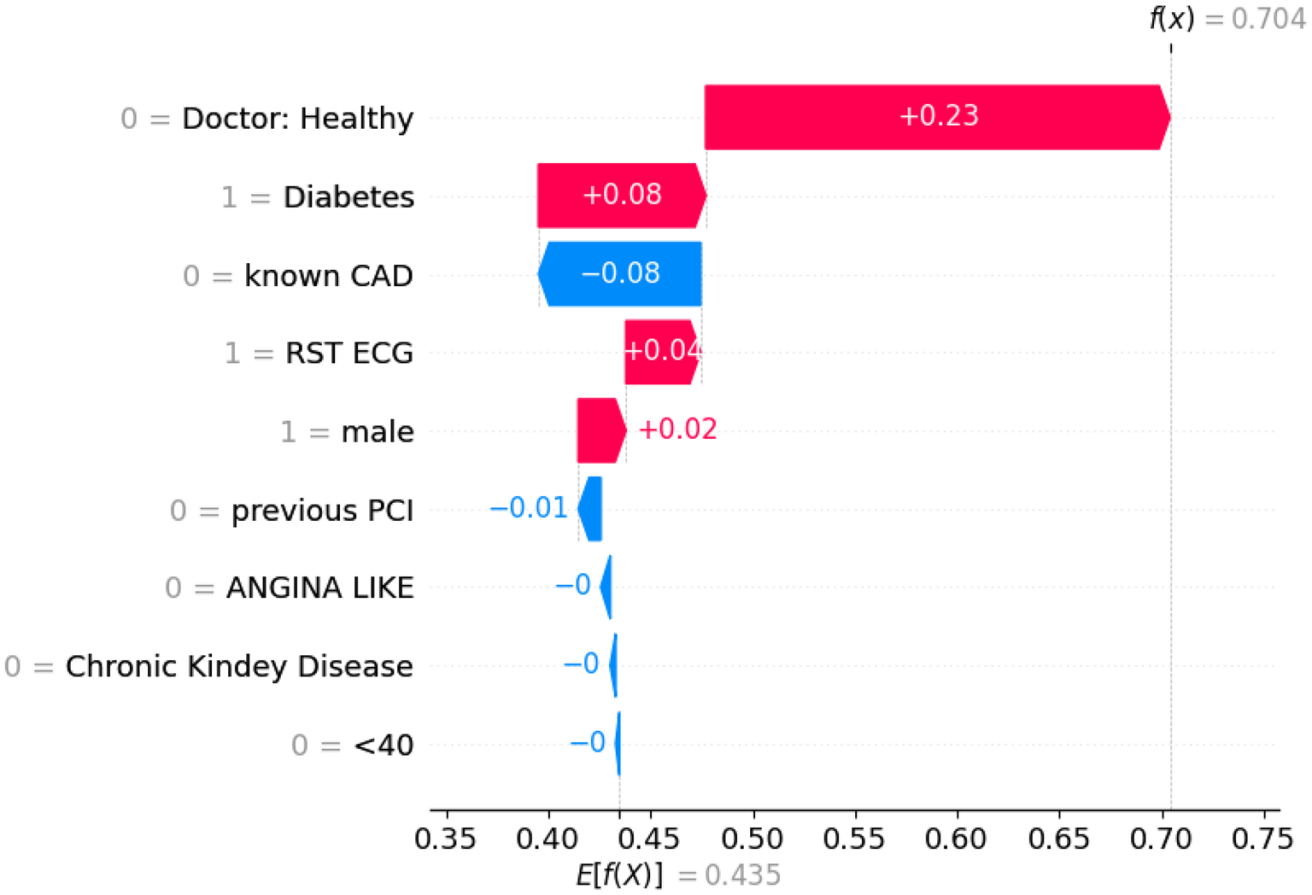
Waterfall diagram for a prediction of a CAD positive patient. This diagram indicates which features drove the algorithm to predict the entry as CAD. The expert had correctly identified this as a CAD instance and ICA confirmed it.

The best performing model for this set of experiments was the one based on the Random Forest algorithm. It achieved an accuracy score of 83.02% and the feature subset that contributed to this result is shown in Table 6. Figure 1 depicts the Cohen effect sizes for each feature in the selected subset, and Fig. 2 displays the relative summary plot using the SHAP values for every feature used as input. Moreover, Fig. 3 showcases the waterfall plot for a CAD positive data entry (unhealthy), while Fig. 4 shows the corresponding waterfall plot for a CAD negative data entry (healthy).

In Fig. 1 the features are ranked based on their impact on the prediction result. The higher the impact, the higher the feature appears in the plot. In other words, the doctor’s initial diagnosis has the highest impact on the prediction result, whereas the feature “Chronic Kidney Disease” the lowest. Positive Cohen effect values indicate the feature leads the prediction to a healthy result. For instance, when the doctor diagnoses the subject as healthy, it leads to a prediction of healthy.

Similarly, in the summary plot of SHAP values (Fig. 2), the features are also sorted in order of magnitude of effect. This summary plot depicts the relative importance of each input feature in the prediction model. The SHAP values for this particular scenario are depicted on the horizontal axis. In our case, negative SHAP values indicate that the feature leads the prediction towards the CAD class (unhealthy subject). For instance, the feature “Doctor: Healthy” has a high negative SHAP value when its value is low (Doctor: Healthy = 0). This indicates that when this feature has a value of ‘1’ (which means the doctor initially predicted the subject was healthy), it leads to a NO-CAD (healthy) prediction. Alternatively, low values of the feature “Diabetes” have high positive SHAP values, indicating that a non-diabetic subject is more likely to lead to a healthy prediction.

As far as the waterfall plots are concerned (Figs. 3, 4), *E[f(x)]* is the prediction threshold and *f(x)* is the model’s prediction result. The decision threshold for this scenario is **0.435** and it is used to label each prediction. Results with a score f(x) lower than the decision threshold E[f(x)] are categorized as NO-CAD (healthy), while results with a score higher than the decision threshold E[f(x)] are categorized as CAD (unhealthy). Ergo, each feature, depending on its value, “pushes” the decision result in the appropriate direction. For instance, in Fig. 3 feature “Doctor: Healthy” has a value of ‘0’ (unhealthy diagnosis) and thus pushes the decision towards an unhealthy outcome (‘1’).

## Discussion

The driving objective behind this work is multifaceted. First of all, a man-in-the-loop approach was to be opted for, which meant taking into account the expert’s prediction in order to explore the benefits of doctor’s prediction in the overall prediction model. Furthermore, feature selection mechanisms were employed to assess the importance of focused datasets in this problem of CAD prediction.

The results of the previous section highlight some major conclusions, both for each case individually and in their combinations. The insight derived from the results can be summed up in the following points:

(i) Feature selection greatly enhanced the performance of all ML prediction models used in this work
(ii) The expert’s verdict can be solidly enhanced with the aid of an ML prediction model
(iii) A prediction model based entirely on clinical data and without the expert’s opinion as input cannot (easily) outperform the doctor/expert
(iv) The feature selection process and the best resulting feature subsets highlight the importance of specific data, as they were present in the majority of selected subsets
(v) Explainability provided valuable insight into the decision-making process of the model, making it more transparent and trustworthy.

Considering every metric, when using as input a more stripped-down dataset with no irrelevant or trivial information, all the ML algorithms performed significantly better. Specifically, accuracy-wise, the results were more accurate by 1.93% for SVM, 10.86% for Decision Tree, 3.67% for K-Nearest Neighbor, 4.02% for AdaBoost and 5.79% for Random Forest. Consequently, comparing the performance of ML models with and without feature selection clearly highlights a performance gap.

The second point highlighted by the results of this work is that a computer-aided decision-making tool/system can surely enhance the prediction accuracy of human experts. Specifically, the expert’s prediction accuracy is 78.81%. As Table 3 exhibits, all the ML prediction models managed to surpass that accuracy by varying margins. Moreover, the model based on random forest, which was the best performing, achieved an accuracy score of 83.02%. Consequently, the utilization of RF improves the expert’s prediction by 4.21%.

Another factor that has a substantial impact on the prediction model is the use of the expert’s opinion itself as an input. Table 7 depicts the best results for every model when employing data preprocessing (feature selection) and not taking into consideration the expert’s verdict. It can be observed that no model managed to achieve accuracy higher than 78.81% which is the accuracy of the expert. Additionally, a comparison with Table 5 highlights the increase in performance when using the expert’s opinion: 4.37% for SVM, 3.67% for Decision Tree, 6.12% for K-Nearest Neighbor, 5.08% for AdaBoost and 5.43% for Random Forest. Given that the expert's/ doctor's forecast is based on years of theoretical and practical expertise, as well as in findings on SPECT/PET imaging data, these outcomes are completely rational in retrospect. Therefore, it is not a simple task to replace the expert's knowledge with an AI prediction model that utilizes mostly clinical and demographic data as inputs. This also justifies some calls in the research community^21,22^ for the inclusion of the doctor in the decision-making loop of AI systems.

**Figure 5:**
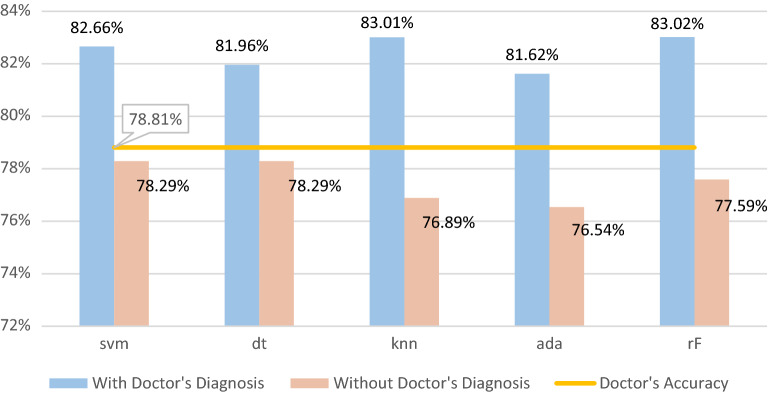
Best tenfold testing accuracies (%) achieved by each ML model when using expert’s verdict as input (blue) and when not (orange). The comparison horizontal line depicts the doctor’s accuracy (78.81%).

Figure 5 clearly illustrates the two arguments above. It can be plainly seen that all the models surpassed the doctor’s accuracy, when using the expert’s diagnosis as input. On the other hand, none surpassed the doctor’s accuracy without the expert’s diagnosis as input.

Considering related studies on the same subject, as displayed in Table 1, some comparisons can be drawn. It is evident that the current study differs from most of the related work in terms of its approach, specifically in the incorporation of expert's opinion as input and the use of interpretability mechanisms to increase transparency and understanding of the model's decision-making process. Almost every other study that focuses on the problem of CAD/NO-CAD classification, does not use the medical expert’s diagnosis as input. On the contrary, this information is used as the testing/labeling case (desired output to be predicted). Ergo, it is unsafe to make direct comparisons of the models’ performance, as the desired prediction output of the current work is confirmed against the actual results of the ICA procedure. Nevertheless, the performance of all prediction models when including the doctor’s yield in their input, as it is presented in the “Results” section, is on par with the body of work in this specific field. Moreover, the dataset used in the current work is one of the largest used in research on this specific scenario. However, there has been a similar study^20^ employing a doctor-in-the-loop approach. The researchers explore the effect of common risk factors for CAD and combine the doctor’s diagnosis as input, similarly to this work. Still, there are some major differences. First and foremost, ICA, which the current paper uses as a test variable for the prediction results, is the gold standard for the diagnosis of obstructive CAD, making the current results and metrics the most reliable. Furthermore, in Benjamins et al.^20^, the expert’s input is in fact the doctor’s interpretation of the Computed Tomography Angiography (CTA) results, whereas in the current study it is taken prior to ICA and based on clinical data. Consequently, in contrast with the method used in Ref.^20^, the prediction models presented in this work do not require a Tomography Angiography procedure for full functionality. Nevertheless, both studies showcase a consistent increase in the accuracy of the model when employing the expert’s yield (in any form).

Furthermore, it is worth mentioning that some of the features were nearly constantly present in the optimal feature subsets for each model. An overview of Tables 6 and 8 indicates that features 'known CAD', 'Diabetes', 'male', ' < 40' were present in every subset, fields 'ANGINA LIKE' and 'RST ECG' in nearly all, and the field 'Chronic Kidney Disease' was also very common. These are very well known indicating risk-factors when studying a patient for CAD^45–52^. However, an unexpected result was that other extremely common CAD risk-factors were absent. In particular, smoking was only included in 2 subsets, whereas Dyslipidemia was included in only two, as well. On the other hand, even though Arterial Hypertension was present in almost every subset when taking the expert’s opinion into consideration, it was not included in any of the subsets when not having the doctor’s prediction as input whatsoever. This is compelling because the doctor’s opinion would be based most probably on this risk factor, thus one would assume that the factor’s absence in the subsets of Table 6 is reasonable. The same, though, cannot be said for the feature subsets of Table 8.

On the matter of explainability, there is a noticeable similarity between the features identified as most impactful through the use of Cohen effect sizes (Fig. 1) and SHAP values (Fig. 2). Additionally, it is immediately evident that the feature with the greatest impact is the doctor’s initial diagnosis. This is a strong argument for the doctor-in-the-loop approach, and it is also quite logical, given the fact that the doctor’s opinion in a sense encapsulates all the other features as well. As displayed in Fig. 2, there is evidently a strong connection between feature “Doctor: Healthy” (i.e. healthy diagnosis) and the final prediction; when the expert diagnoses the entry as healthy it leads to a prediction of NO-CAD (healthy). Another major factor is, apparently, the history of CAD for the patient. If the patient was CAD affected in the past, it is very probable that they will be CAD positive. There is also a correlation between CAD and Diabetes (when a subject is diabetic it leads to an unhealthy prediction), which is a well-documented risk factor for CAD. Males also seem to be more prone to CAD than females and subjects who lead more active lifestyles (high values of RST ECG) are less prone to be CAD affected. All in all, both the Cohen effect size chart and the SHAP summary plot are quite consistent with medical bibliography^45–52^ on the subject of cardiovascular diseases and its common risk factors.

Figures 3 and 4 showcase the decision process of the best-performing prediction model for a healthy subject (Fig. 3) and a CAD positive one (Fig. 4). More specifically, in the case of the healthy subject, the initial expert diagnosis was that the subject had CAD (but the ICA results later proved otherwise). As such, this outlier case greatly impacts the performance of the prediction model. However, taking into account the other contributing factors such as the patient's lack of a CAD history and diabetes, the model ultimately reached the correct conclusion that the subject does not have CAD (score threshold for CAD: **0.435**; entry’s score: **0.39**). On the other hand, as the waterfall plot in Fig. 4 showcases, this prediction was much clearer. Specifically, the expert had correctly diagnosed the patient as CAD positive, thus greatly influencing the model towards this notion. The patient had no history of CAD; he/she was, however, diabetic. Overall, this particular entry’s score was **0.704** against an effective threshold of **0.435**; thus, the model successfully identified this instance as a CAD positive one.

In conclusion, the results of this study highlight the significance of explainability in machine learning models for CAD diagnosis. The use of explainability mechanisms such as Cohen effect sizes and SHAP values provide insight into the decision-making process of the model and allows for better understanding of the factors that contribute to its predictions. This can increase the trust and acceptability of the model among experts and healthcare professionals. Additionally, the explainability insight provided by this study also allows for better identification of outliers and potential errors in the input data, leading to more accurate predictions and improved diagnosis of coronary artery disease.

Nevertheless, this study has some limitations. In the scope of this study, only clinical data were used, without any image data from SPECT/PET. It is most likely that the addition of these images might further improve the prediction accuracy of the models. Moreover, the ML models presented in this work function as black boxes. That is, there is no direct insight into the weights and leading factors used by the algorithms when making the prediction of whether a patient is CAD-affected or not.

## Conclusion

The main objective of this work was to assess the impact of a man-in the-loop approach to the CAD prediction problem. It is shown that the ML models manage to integrate the human expert’s yield and improve the diagnostic accuracy by 5% when using the human expert in the loop. This work has highlighted the importance of an AI aided decision-making tool in the process of CAD evaluation. The results have showcased a substantial enhancement of prediction accuracy, when including the expert’s opinion on the input data. It has also been pointed out that feature selection can lead to further improvement of the prediction results for the ML models. Involving the expert in the decision/prediction making process is also emphasized, as this has a significant impact on the accuracy of the final prediction. Interestingly, some common risk factors for CAD (e.g. Dyslipidemia) were consistently ignored during the feature selection process. In the future, we plan to incorporate image data or prediction results from AI models that utilize image data as inputs. Furthermore, more research could be conducted on the findings of Sect. 2, specifically on these well-documented CAD risk factors that were not selected in the feature subsets, such as smoking and arterial hypertension.

## Data Availability

The datasets used and/or analyzed during the current study are available from the corresponding author on reasonable request. The repo containing the scripts that were used to produce the above findings is located at: https://github.com/agosamaras/CAD-AI.
